# Pharmacological Modulation and (Patho)Physiological Roles of TRPM4 Channel—Part 2: TRPM4 in Health and Disease

**DOI:** 10.3390/ph15010040

**Published:** 2021-12-28

**Authors:** Csaba Dienes, Zsigmond Máté Kovács, Tamás Hézső, János Almássy, János Magyar, Tamás Bányász, Péter P. Nánási, Balázs Horváth, Norbert Szentandrássy

**Affiliations:** 1 Department of Physiology, Faculty of Medicine, University of Debrecen, 4032 Debrecen, Hungary; dienes.csaba@med.unideb.hu (C.D.); kovacs.zsigmond@med.unideb.hu (Z.M.K.); hezso.tamas@med.unideb.hu (T.H.); almassy.janos@med.unideb.hu (J.A.); magyar.janos@med.unideb.hu (J.M.); banyasz.tamas@med.unideb.hu (T.B.); nanasi.peter@med.unideb.hu (P.P.N.); horvath.balazs@med.unideb.hu (B.H.); 2 Doctoral School of Molecular Medicine, University of Debrecen, 4032 Debrecen, Hungary; 3 Division of Sport Physiology, Department of Physiology, Faculty of Medicine, University of Debrecen, 4032 Debrecen, Hungary; 4 Department of Dental Physiology and Pharmacology, Faculty of Dentistry, University of Debrecen, 4032 Debrecen, Hungary; 5 Faculty of Pharmacy, University of Debrecen, 4032 Debrecen, Hungary; 6 Department of Basic Medical Sciences, Faculty of Dentistry, University of Debrecen, 4032 Debrecen, Hungary

**Keywords:** TRPM4, SUR1, pancreatic β-cell, cancer, neuronal, immune system, urinary bladder, cardiac, vascular tone, central nervous system injury

## Abstract

Transient receptor potential melastatin 4 (TRPM4) is a unique member of the TRPM protein family and, similarly to TRPM5, is Ca^2+^ sensitive and permeable for monovalent but not divalent cations. It is widely expressed in many organs and is involved in several functions; it regulates membrane potential and Ca^2+^ homeostasis in both excitable and non-excitable cells. This part of the review discusses the currently available knowledge about the physiological and pathophysiological roles of TRPM4 in various tissues. These include the physiological functions of TRPM4 in the cells of the Langerhans islets of the pancreas, in various immune functions, in the regulation of vascular tone, in respiratory and other neuronal activities, in chemosensation, and in renal and cardiac physiology. TRPM4 contributes to pathological conditions such as overactive bladder, endothelial dysfunction, various types of malignant diseases and central nervous system conditions including stroke and injuries as well as in cardiac conditions such as arrhythmias, hypertrophy, and ischemia-reperfusion injuries. TRPM4 claims more and more attention and is likely to be the topic of research in the future.

## 1. Introduction

Transient receptor potential (TRP) channels were discovered in Drosophila when the structure of a protein fulfilling a role in phototransduction was described in 1989 [1]. The 28 members of the TRP family are divided into six subfamilies based on the sequence homology among the members. These include TRP Canonical (TRPC1-7), TRP Vanilloid (TRPV1-6), TRP Melastatin (TRPM1-8), TRP Ankyrin 1 (TRPA1), TRP Mucolipin (TRPML1-3), and TRP Polycystin (TRPP2, TRPP3, and TRPP5) [2]. The subfamily of transient receptor potential melastatin (TRPM) consists of eight members (TRPM1-8), forming four pairs based on the similarity of their sequences [3]. A pair is made of TRPM4 and TRPM5, which are unique as they are only permeable to monovalent but not divalent cations [4,5]. Ion substitution experiments have revealed that the selectivity sequence of Na^+^ > K^+^ > Cs^+^ > Li^+^ for TRPM4 [6,7]. This channel has a widespread tissue expression [8] and is activated by intracellular Ca^2+^ [9,10].

TRPM4 couples the increase of intracellular calcium levels to changes in the membrane potential. Due to this coupling, TRPM4 fulfills many roles in both excitable as well as non-excitable cell types. TRPM4 mutations are linked to cardiac conduction problems such as atrioventricular conduction block, Brugada syndrome, right bundle branch block, and congenital long QT syndrome [11,12]. TRPM4 also contributes to insulin secretion by pancreatic cells [13,14], immune cell activity [15], and plays a role in cancer [16,17].

This part of the review summarizes the current state of knowledge on the pathological and physiological roles of TRPM4. Part 1 of the review discusses the modulation of TRPM4 by both endogenous and exogenous activators and inhibitors of the ion channel, as well as molecular biological approaches to studying the role of TRPM4.

## 2. Physiological Roles of TRPM4

### 2.1. Presence of TRPM4 in Various Cells/Organs

Even before the discovery of TRPM4, which is the ion channel responsible for the calcium-activated monovalent cation currents, several reports suggested a role for these currents, for instance in neonatal rat cardiac cells [18] and outer hair cells of guinea pig cochlea [19]. Since that time, TRPM4 expression has been confirmed in several tissues and cell types and a potential role for TRPM4 has been suggested. The smooth muscle cells [20] of the vasculature [21,22,23], the urinary bladder [24,25,26,27], and the colon [28]; the endothelium [29,30]; immune cells [15,31]; several parts of the central nervous system [32]; endocrine cells of the islets of Langerhans [14,33]; adipocytes [34]; keratinocytes [35]; mucin secreting cells [36]; human valve interstitial cells [37]; and skeletal muscle [8] all express TRPM4. TRPM4 was implicated in various types of cancer [38,39]. TRPM4 expression is also pronounced in the heart and the channel can contribute to the function of various types of cardiac cells including sinoatrial nodal cells [10,40], atrioventricular nodal cells [12], cells of the conduction system [11,41], and atrial [4,42] and ventricular myocytes [43,44].

### 2.2. Physiological Importance of TRPM4

#### 2.2.1. The Role of TRPM4 in the Secretory Mechanism of the Endocrine Pancreas

TRPM4 can be involved in the function of islets of Langerhans [14]. A Ca^2+^-activated non-specific cationic current (I(NSC_Ca_)) was described before the discovery of TRPM4. A current inhibited by adenosine triphosphate (ATP) was described in CRI-G1 rat insulinoma cells [45] and in β-cells of human and murine islets [46]. As phosphatidylinositol 4,5-bisphosphate (PIP2) effectively facilitates the activation of TRPM4 and the PIP2 level increases upon the activation of β-cells by glucose, it is tempting to speculate that TRPM4 is indeed important to the function of these cells. Furthermore, as β-cells are the targets of the antidiabetic drug glibenclamide, and it was shown that glibenclamide inhibits TRPM4, this also suggests a role for TRPM4. Indeed, TRPM4 RNA is present in several β-cell lines such as HIT-T15 (hamster), RINm5F (rat), β-TC3, and MIN-6 (mouse) [47]. Not only was the presence of TRPM4 mRNA confirmed, but a TRPM4 protein was also shown in RINm5F and in INS-1 rat β-cell lines [13] and in human pancreatic islets [47]. In these cell lines, the glucose- and arginine vasopressin-induced stimulation led to depolarization and insulin secretion with two phases. The first phase was due to the activation of the TRPM4 channels and the second, slower phase was due to the recruitment of TRPM4-containing vesicles to the plasma membrane [13]. In cells expressing the dominant negative TRPM4 construct, depolarization and insulin secretion were much smaller. Similarly, two phases of depolarization and insulin secretion were induced in the β-cells of other species in response to various stimuli, including glibenclamide on top of glucose and arginine vasopressin [47]. Not only was the dominant negative construct effective in reducing the stimulation-induced insulin secretion, but 20 and 30 µM 9-phenanthrol also reduced insulin secretion induced by glucose and GLP-1 in isolated rat islets [48]. Similarly, GLP-1-induced insulin secretion—mediated by Protein Kinase C (PKC) and the mobilization of intracellular Ca^2+^ from thapsigargin-sensitive Ca^2+^ stores—involved TRPM4 activation, as it was negligible in the TRPM4 knock-out (KO) mice [49]. Pancreatic α-cells (α-TC1-6 murine cells) also express TRPM4 both at the RNA and protein levels, and TRPM4 contributes to agonist-induced depolarization and glucagon secretion as TRPM4 knockdown significantly reduced both depolarization and glucagon secretion upon stimulation with arginine vasopressin, KCl, L-arginine, and BayK 8644 [50]. Similarly, the hamster α-cell line INR1G9 also functionally expressed TRPM4, but, unlike in β-cells, no second phase was observed [47]. Although several studies indicate that TRPM4 has a role in insulin secretion, it must be noted that TRPM4 KO animals do not have hyperglycemia, insulin deficiency, or diabetes mellitus, so further studies are required to elucidate the physiological role of TRPM4 in the function of the endocrine pancreas.

Murine non-cardiac TRPM4 KO animal models are summarized in Table 1.

#### 2.2.2. The Role of TRPM4 in the Immune System

TRPM4 was also connected to the function of various immune cells. Although the presence of TRPM4 RNA was not detected in human spleen, thymus, or lymphocytes [57]; TRPM4 protein was present in mouse thymocytes, in D10.G4 cells (murine Th2 cell clone), in Molt-4, and Jurkat cells (human T lymphoblast cell lines) [31]. TRPM4 might play a negative feedback role in the activation of immune cells as an expression of the dominant negative TRPM4 construct, and the small-interfering RNA (siRNA)-mediated TRPM4 silencing converted the phytohemagglutinin-induced intracellular Ca^2+^ oscillations to a sustained Ca^2+^ concentration increase. Reduction of TRPM4 currents led to an increase in IL-2 production [31]. TRPM4 activation would lead to depolarization, which, by reducing the driving force for Ca^2+^ entry and acting in concert with other channels, can control Ca^2+^ oscillations in lymphocytes. A similar negative feedback role for TRPM4 was suggested in stimulated mast cells [15]. Ca^2+^ signals, the release of histamine, leukotrienes, and tumor necrosis factor, as well as IgE-mediated cutaneous acute—but not chronic—phase allergic reactions, were all augmented in TRPM4 KO mice [15]. TRPM4 contributed to the migration but not to the maturation of murine dendritic cells by different mechanisms [58]. Migration of bone-marrow-derived mast cells was also reduced in TRPM4 KO mice [56]. TRPM4 expression was higher in murine Th2 cells compared with Th1 ones and differentially regulated Ca^2+^ signaling and the nuclear factor of activated T-cells (NFATc1) localization [59]. The reduction of TRPM4 expression by siRNA led to an increased Ca^2+^ influx and oscillatory levels in Th2 cells but the opposite effect was observed in Th1 cells. Furthermore, TRPM4 silencing reduced cell velocity and motility in Th2 cells but increased these functions in Th1 cells [59]. TRPM4 function improved survival in a murine sepsis model by facilitating monocyte and macrophage, but not neutrophil, functions including AKT signaling pathway-mediated phagocytic activity [55]. The compound 9-phenanthrol induced the degranulation of mast cells in vitro, although not due to TRPM4 inhibition, but likely by activating type 3.1 Ca^2+^-activated K^+^ channels [60]. Nevertheless, 9-phenanthrol used in combination with imiquimod in a murine tumor model enhanced the in vivo degranulation of mast cells, the migration of dendritic cells, and the expansion of antigen-specific cytotoxic T lymphocytes thereby improving the efficiency of prophylactic immunization [60]. TRPM4 is involved in the function of several types of immune cells, at least in mice. TRPM4′s mRNA level was smaller in peripheral blood-derived mononuclear cells of patients suffering from psoriasis than in cells from healthy subjects, indicating the possible role of TRPM4 in the pathogenesis of psoriasis [61]. A recent article described TRPM4 as the target of protein arginine methyltransferase 5-mediated alternative splicing in activated murine T cells [62].

#### 2.2.3. The Role of TRPM4 in the Regulation of Vascular Tone

TRPM4 has been extensively studied in vascular smooth muscles. One report mentioned a current activated by noradrenalin having permeability for monovalent cations in smooth muscle cells of rabbit ear arteries [63]. TRPM4 RNA was shown in whole rat cerebral arteries, in smooth muscle cells isolated from those vessels [64], and also in rat aorta and pulmonary vessels [65]. Smooth muscle cells isolated from cerebral arteries express the TRPM4 protein [66,67]. In rat cerebral arteries, TRPM4 mediated pressure-induced myogenic vasoconstriction [64] in a PKC-regulated manner [68]. Additionally, basal PKCδ activity increased the cell surface expression of TRPM4, thereby enabling TRPM4-mediated vasoconstriction [66,69]. In these studies, TRPM4 antisense oligodeoxynucleotides applied with reverse permeabilization technique on isolated arteries proved the role of TRPM4. The use of TRPM4 antisense oligodeoxynucleotides confirmed the role of TRPM4 in in vivo tests, as the suppression of TRPM4 led to decreased cerebral artery myogenic constrictions and impaired autoregulation [70]. In rat cerebral arterial myocytes, TRPM4 could contribute to membrane stretch-induced depolarization [71]; although the direct stretch sensitivity of TRPM4 was not confirmed, and it is likely that such a stretch leads to a calcium increase and TRPM4 activation. Furthermore, the functional cooperation between TRPC6 and TRPM4 was indicated [72], where the stretch sensor type 1 angiotensin II receptor activates phospholipase Cγ1 by Src tyrosine kinase, leading to diacylglycerol and inositol 1,4,5-trisphosphate (IP3) formation. The stretch activates the TRPC6, which, upon opening, leads to a Ca^2+^ influx and further activation of the IP3 receptor. The IP3 receptor co-localizes with TRPM4 in nanodomains [73], and the release of Ca^2+^ through the IP3 receptor was shown to activate TRPM4 [67], leading to depolarization and activation of voltage gated calcium channels, Ca^2+^ influx, and smooth muscle contraction. Supporting the functional cooperation, both 9-phenanthrol and the TRPC6 inhibitor larixyl acetate reduced the myogenic tone in mesenteric arteries (developed due to either KO of elastin microfibrils interface located protein-1 or the transforming growth factor β (TGF-β) treatment of wild type mice) [74]. The role of TRPM4 in the maintenance of vascular tone is supported by another study, wherein the TRPM4 inhibitor 9-phenanthrol led to the hyperpolarization of the smooth muscle cells and vasodilation [75]. Finally, TRPM4 was implicated in the coupling of P2Y4 and P2Y6 receptor-mediated mechanoactivation and myogenic tone development in cerebral parenchymal arterioles. Later on, the involvement of RhoA/Rho-associated protein kinase signaling was confirmed in the process, as the Rho-associated protein kinase inhibitor H1152 strongly attenuated, while the RhoA activator CN03 potentiated the pressure-induced constriction [76]. TRPM4 seems to be involved in the regulation of arterial tone at least in the cerebral vessels of rats, despite the lack of difference in agonist- or pressure-induced contractile responses detected in the aorta or in hindlimb preparations isolated from wild-type or TRPM4 KO mice [77]. One might argue that the different species are the reason for the discrepancy. Moreover, the systemic KO of TRPM4 can lead to compensatory changes. It must be noted that TRPM4 KO mice were mildly hypertensive, due to the slightly increased catecholamine secretion, which can compensate for the loss of TRPM4-induced smooth muscle contractility. Unfortunately, the reports from rat TRPM4 KO animals [78,79] do not describe vascular smooth muscle function. In murine cerebral arterial smooth muscle cells, TRPM4 channels are responsible for nitric oxide-mediated vasodilation via inhibition of the PKG substrate-mediated, IP3 receptor-dependent Ca^2+^ release [80].

#### 2.2.4. The Role of TRPM4 in Chemosensation

TRPM5 is generally accepted to be involved in the olfactory system [81]. TRPM4 is expressed in murine accessory olfactory bulb mitral cells, where it mediates, at least in part, the sustained responses evoked by the calcium-activated nonselective cationic current [82]. A calcium-activated nonselective cationic current is present in the vomeronasal sensory neurons of hamsters, where it can either directly mediate vomeronasal sensory transduction or amplify the primary sensory response [83]. In mice, the TRPM4 protein was itself expressed in vomeronasal sensory neurons where its expression was estrous cycle-dependent, suggesting TRPM4 participation in the sex-specific, estrous cycle-dependent, and sex hormone-regulated functions of the vomeronasal organ [84].

TRPM5 is clearly important to taste sensation [85], but the contribution of TRPM4 in sensing sweet, bitter, and umami tastes in mice has also been suggested [51]. The TRPM4 protein was expressed in type II and type III but not type I taste cells, whereas its absence in the KO mice strain resulted in significantly impaired taste sensation. TRPM4 and TRPM5 are the only two channels mediating taste-induced Na^+^ entry; complete loss of taste sensation was only detected in the double (TRPM4 and TRPM5) KO mice indicating the requirement of not only TRPM5 but also TRPM4 for sensation of sweet, bitter, and umami tastes [51].

#### 2.2.5. The Role of TRPM4 in Renal Physiology

TRPM4 mRNA expression was weak in murine kidneys [86,87], but it was detected in rat [88] and human kidneys [8]. A TRPM4-like current was described in the M-1 mouse cortical-collecting duct cell line [89] and basolateral membrane fragments of murine cortical thick ascending limbs cells [90], as well as in cells of other tubular segments, including proximal (pars recta), distal convoluted, connecting and outer medullary collecting tubules, the thin descending limb, and the medullary thick ascending limb [91]. Calcium sensitivity was highest in the thin descending limb, high in the cortical thick ascending limb, lower in the medullary portions of the thick ascending limb and distal convoluted tubule, and lowest in the connecting tubule and the cortical and medullary collecting tubule. A Ca^2+^-activated, non-selective current permeable for monovalent cations and inhibited by ATP, ADP, and AMP was present in the apical membrane of rat inner medullary collecting duct cells [92]. Renal ischemia-reperfusion reduced TRPM4 mRNA in rats [88]. A TRPM4-mediated current was detected on native cilia of renal epithelial murine cell line [93]. TRPM4 silencing led to a reduction in cilia length; the percentage of cells with cilia in the cell culture and TRPM4 might influence the apical Ca^2+^ dynamics of these cells [93]. Finally, TRPM4 protein was detected in the mpkCCDc14 line (an immortalized mouse collecting duct principal cell), where H_2_O_2_ suppresses its trafficking to the apical membrane [94]. These results indicate that the previously mentioned non-selective current is indeed likely to be mediated by TRPM4, although its role in renal physiology is still not known.

#### 2.2.6. The Role of TRPM4 in Respiratory Neuronal Activity

TRPM4 can contribute to the activity of neurons controlling respiration. Flufenamic acid reduced the bursting activity of neurons in the pre-Bötzinger complex slice preparations obtained from neonatal mice, suggesting a role for a Ca^2+^-activated inward cationic currents, probably mediated by TRPM4 [95]. Indeed, TRPM4 mRNA-expressing neurons were described in the pre-Bötzinger complex region of neonatal mice [96], and the TRPM4 protein was present in neurons of the pre-Bötzinger complex of the rat [97]. TRPM4 channels were modulated by calcium, calmodulin, and ADP in the pre-Bötzinger complex neurons in functionally intact slices and may become more important in times of increased respiratory activity such as hypoxia [98]. The mRNA and protein expressions of both TRPM4 and TRPC3 were shown in functionally identified glutamatergic, glycinergic, and GABAergic inspiratory pre-Bötzinger complex neurons as well as inspiratory motoneurons [99]. In addition to endogenously activated TRPM4 (likely mediating Ca^2+^-activated inward cationic current), TRPC3 was also implicated as playing an essential role in respiratory pattern formation, but not in respiratory rhythm generation in both neonatal and mature rodents [99]. Some of these results were confirmed in adult mice using TRPM4-targeted short hairpin RNA, where TRPM4 suppression decreased the tidal volume but increased respiratory frequency, often followed by gasping and fatal respiratory failure [100]. The contribution of TRPC3 was questionable, which was, in all likelihood, due to the different concentrations and off-target actions of the TRPC3 inhibitor used in the two studies. Recently, TRPM4 was detected on neurons of the retrotrapezoid nucleus of the mouse and the channel was involved in large-amplitude membrane potential oscillations suggesting a role for TRPM4 in pacemaker-like firing required for basal, CO_2_-stimulated, and state-dependent breathing [101].

#### 2.2.7. The Proposed Role of TRPM4 in Various Other Neuronal Function

Apart from the previously mentioned role in respiratory centers, TRPM4 can be involved in other neuronal functions. TRPM4 immunoreactivity occurred in cell bodies of both vasopressin and oxytocin neurons [102]. In murine cerebellar Purkinje cells, TRPM4 (and also TPRM5) can be involved (but not be exclusively responsible) in generating a strong depolarization-induced slow, inward cation current [103]. Moreover, TRPM4 can fine-tune the Ca^2+^ influx and thereby influence the firing activity of rat cerebellar granule cells [104]. TRPM4 can contribute to the persistent firing pattern of murine thalamic reticular neurons [105]. In murine CA1 pyramidal neurons, TRPM4 was only expressed on the soma at birth and its expression extended during postnatal development from the soma to the apical dendrites but not to the axon initial segment [106]. A TRPM4-like current was active at the resting membrane potential from birth and increased during development, but 10 µM 9-phenanthrol-induced current inhibition hyperpolarized the membrane potential only at later stages. TRPM4 channels are open at the resting membrane potential of the murine prefrontal cortex layer; 2/3 pyramidal neurons and their inhibition increase input resistance and hyperpolarize the membrane potential, thereby modulating neuronal excitability in a calcium-dependent manner [107]. The involvement of TRPM4 in N-methyl-d-aspartic acid (NMDA) and the receptor-dependent long-term potentiation in CA1 hippocampal neurons was also reported [108]. TRPM4, in cooperation with NMDA receptors, can be involved in the generation of tonic and bursting activity of dopaminergic neurons in pars compacta of substantia nigra [109]. TRPM4 can also contribute to subthreshold oscillations in neurons of murine locus coeruleus and suprachiasmatic nuclei [110].

The properties of rat TRPM4 KO animal models are summarized in Table 2.

#### 2.2.8. TRPM4 in the Heart

As shown before in many tissues, first the characterization of I(NSC_Ca_) was done. After the discovery of TRPM4 in many cases, it emerged as the most likely candidate for the ion channel responsible for I(NSC_Ca_). Several reviews detail the contribution of TRP channels to cardiovascular functions [112]. Some focus specifically on TRPM4 [113,114,115]. TRPM4 became an important player in cardiac electrical activity [116] and was involved in pathological conditions, as will be mentioned later.

##### The Role of TRPM4 in Pacemaking

TRPM4 exists in nodal tissues, because TRPM4 mRNA [117] and protein [10] were detected in mice. Ion currents with TRPM4-like characteristics were detected not only in mice [10], but also in rats [40] and rabbits [40,118]. TRPM4 inhibition, achieved by 9-phenanthrol, led to a dose-dependent reduction of heart rate in wild type but not in TRPM4 KO mice, supporting an important role of TRPM4 in pacemaking [40]. It must be noted, however, that the heart rate was not reduced in TRPM KO animals compared with wild type ones [77,117,119]. These observations argue against the role of TRPM4 in pacemaking or might be the consequence of compensatory changes in the KO animals. As pacemaking is an essential function, it is highly protected by the cooperation of several mechanisms (Ca^2+^ and membrane clock theories) [120]. Therefore, it is possible that, in the case of TRPM4 KO animals, the contribution of other non TRPM4-mediated mechanisms is modified in such a way that the heart rate eventually remains unchanged. On the contrary, the heart rate was significantly lower in TRPM4 KO mice but only on the surface and not the pseudo ECG [121]. In case of rabbits, 9-phenanthrol slightly but significantly reduced the spontaneous beating rate and the rate of diastolic depolarization without modifying any other AP parameters on isolated sinoatrial cells [40]. Despite these results, several questions remain to be answered as suggested by a short review [122].

##### The Contribution of TRPM4 to Atrial Electrophysiology

Ca^2+^-activated non-selective cation currents were reported in neonatal rat atrial myocytes [123] and isolated atrial cells from right atrial appendages [4]. Moreover, TRPM4 mRNA was detected in human atrial muscle [4]. TRPM4 protein expression was similar in human myocytes isolated from right atrial appendages of individuals both with and without atrial fibrillation [124]. The function of TRPM4 in atrial AP was characterized using the combination of TPRM4 KO mice and a pharmacological approach [42]. 9-phenanthrol- or flufenamic acid-induced TRPM4 inhibition reduced the AP duration of isolated atrial cells in a reversible and dose-dependent manner in cells from wild type but not KO animals. Other AP parameters were unaltered (except a small reduction in AP amplitude using a high (100 µM) dose of 9-phenanthrol). On the contrary, atrial AP was not modified by as high as 100 µM 9-phenanthrol when studied in intact mouse right atrial muscles [40]. Nevertheless, atrial APs were shorter in TRPM4 KO animals compared with those of wild type ones [42,117,119], except in a recent report where the only difference was a slightly more depolarized resting membrane potential in the KO animals [121]. In congruence with previous studies where atrial APs were shorter in TRPM4 KO animals, mild (2–5 folds) and large (at least 6 folds) overexpression of TRPM4 using computer simulation led to an increase in AP duration and generation of early afterdepolarizations (EADs) [125]. These simulations were consistent with electrophysiological data obtained from the immortalized rat atrial HL-1 myocytes, where angiotensin II increased the expression of TRPM4 [125]. TRPM4 activity also increased following the functional coupling to CaMKIIδ in HL-1 myocytes [126]. In rat atrial cells, TRPM4-mediated currents could be involved in the response to shear stress via activation of type 2 IP3 receptor-mediated Ca^2+^ releases [127]. TRPM4 can play a role in aldosterone-induced action potential (AP) shortening and may promote aldosterone-induced atrial arrhythmias [119]. TRPM4 was also implicated in the function of human and murine atrial fibroblasts where its expression (both mRNA and protein), as well as the ion current, increased during the culturing process, which contributed to cell growth [128].

##### The Role of TRPM4 in Cardiac Conduction

TRPM4 function in atrioventricular nodal tissue was not reported, but the TRPM4 mRNA level was the highest in the Purkinje cells within the human heart [129]. The TRPM4 protein was highly expressed in the Purkinje cells of the bovine heart [11]. In rabbit Purkinje cells, 9-phenanthrol reversibly and dose-dependently shortened the APs without modifying any other AP parameters [41]. In addition, a TRPM4-like single channel current (conductance of 23.8 pS; equal permeability for Na^+^ and K^+^; sensitivity to voltage, Ca^2+^, and 9-phenanthrol) was recorded in Purkinje cells. During the plateau phases of Purkinje AP, action potential clamp experiments revealed a transient, inward, 9-phenanthrol-sensitive current [41]. These findings indicate the contribution of TRPM4 to the AP characteristics of rabbit Purkinje cells and its potential involvement in cardiac conduction and arrhythmia generation.

Indeed, in human pathology TRPM4 mutations were first reported to be responsible for cardiac conduction disorders [130]. The first identified mutation was an amino acid change in the 7th position from glutamate to lysine (E7K), which leads to gain-of-function by reduced endocytosis and increased TRPM4-current density in the cell membrane of mainly Purkinje fibers [129]. The autosomal-dominant E7K mutation led to a progressive familial heart block type I in 3 branches of a large South African Afrikaner pedigree. The higher PIP2 sensitivity of the E7K mutant compared with wild type TRPM4 can also contribute to the gain-of-function effect [131]. Moreover, in the E7K mutant channels, the open state is much preferred, as it has increased voltage-and Ca^2+^-sensitivities [132]. In computer simulations, increased E7K mutant current density led to resting membrane potential depolarization and AP prolongation. An increase of the E7K mutant (but not wild type) TRPM4-channel density also progressively reduced the AP conduction velocity, eventually culminating in a complete conduction block [132]. Three other point mutations (R164W, A432T, and G844D) with autosomal dominant inheritance in one Lebanese and two French families were associated with gain-of-function by the same mechanism: reduced endocytosis due to deregulation of Small Ubiquitin MOdifier conjugation (SUMOylation) [11]. These mutations did not influence the general biophysical characteristics of the TRPM4 current, except for an increase of Ca^2+^ sensitivity in the case of G844D.

An A432T mutation was also found in congenital and childhood atrioventricular blocks, but, in contrast to the previous study, no evidence for a SUMOylation problem was detected. Moreover, the current density was smaller and not higher than that for wild type TRPM4 [133]. A reason for the difference might be related to the different experimental conditions (recording at room temperature vs. 37 °C), as reduced expression of A432T was rescued by reducing the temperature from 37 to 28 °C [133]. Interestingly, gain-of function was detected for E7K in agreement with the study of Kruse et al. [129] suggesting that temperature sensitivity of expression is different in the case of various TRPM4 mutants. More and more TRPM4 mutations were linked to conduction problems. For instance, 13 rare mutations were detected in a study examining 95 unrelated patients of progressive cardiac conduction disease. The I376T mutation was characterized in detail and resulted in a larger TRPM4 current upon expression in human embryonic kidney (HEK) cells [134]. Another study described 6-point mutations, where 160 unrelated patients with various types of inherited cardiac arrhythmic syndromes were tested. TRPM4 mutations were found in the atrioventricular block and the right-bundle branch block but not in sinus node dysfunction, Brugada, or long QT syndrome [12]. Several TRPM4 polymorphisms, most of them in Brugada syndrome patients, were identified and based on the variable clinical phenotypes of both point mutations and polymorphisms. Stallmeyer et al. concluded that additional factors are also likely to modulate the disease phenotype in some patients [12]. Not only gain-of-function (Q854R) but also loss-of-function mutations (A101T, S1044C, and a double variant, A101T/P1204L) could lead to complete heart block [135]. A novel mutation (R819C) was described in a Chinese family with an atrioventricular block very recently [136]. Another loss-of-function mutation (G858A) was reported in a patient with a left ventricular noncompaction complicated by progressive cardiac conduction defects [137]. The link behind TRPM4 overexpression and the atrioventricular block was established by computational modeling, where the doubling of the TRPM4 currents led to EAD formation. Gaur et al. concluded that TRPM4 channels might be responsible for background sodium current, and heterogeneous TRPM4 expression in the His/Purkinje system led to a type II heart block [138].

TRPM4 mutations were reported in 6% of Brugada syndrome patients [139]. Interestingly, some of these, such as T873I and L1075P, led to gain-of-function but some, such as P779R and K914X, resulted in a reduced TRPM4 current. Any change in the resting membrane potential induced by TRPM4 mutations can reduce sodium-channel availability and contribute to Brugada syndrome. In many cases of Brugada syndrome, a mutation of the SCN5A gene leading to a change in sodium current is the reason of the conduction disorder. Moreover, a study identified the TRPM4 mutation G844D which, upon digenic inheritance with a mutation in SCN5A, was responsible for Brugada syndrome [140]. Loss-of-function TRPM4 mutations, when present in a heterozygous form, were not associated with Brugada syndrome 1 but only in the case of two mutations (compound heterozygous TRPM4 null mutations) led to a lack of TRPM4 current and Brugada syndrome [141]. Very recently, TRPM4 mutations were among the multiple genes associated with Brugada syndrome [142].

Four TRPM4 mutations, but no other mutations of the 13 major long QT syndrome genes were found in approximately 2% of the 178 patient-containing cohort [143]. The TRPM4 mutants, V441M and R499P, were almost absent in control populations, but the mechanism that describes how TRPM4 loss-of-function could lead to long QT syndrome is unknown. A more complex pleiotropic effect than merely action potential alteration was suggested. Mutations of G219E and T160M in KCNQ1 and TRPM4, respectively, were found in a 37-year-old female long QT syndrome Uygur patient [144]. Pluripotent stem cell cardiomyocytes obtained from that patient could recapitulate the electrophysiological features of long QT syndrome in vitro.

A TRPM4 structural variant identified in heterozygous deletion was suggested to be pathogenic in sudden unexplained deaths [145].

The known mutations of TRPM4 leading to cardiac conduction problems are summarized in Table 3.

##### The Contribution of TRPM4 to Ventricular Electrophysiology

The involvement of TRPM4 in ventricular AP and function is the most debated field as early reports indicated a very small expression of TRPM4 in the ventricle compared with other parts of the heart. For instance, the mRNA expression of TRPM4 was lowest in the human left ventricle [129] but was present in the right ventricle of a pediatric patient with tetralogy of Fallot [111]. Although the TRPM4 protein was expressed in murine ventricular muscle and myocytes [121], atrial expression was higher [77]. In rats, however, approximately equal expression was detected in atrial and ventricular myocytes [127]. Early reports detected TRPM4-like currents in cultured, dedifferentiated, but not native rat ventricular cells [146,147]. Moreover, a TRPM4-like current (and mRNA) was hardly detected in the left ventricular cells of Wistar rats but appeared in the left ventricular cells of spontaneously hypertensive rats [43]. Despite these abovementioned results, Ca^2+^-activated non-selective cation currents were reported in cultured rat ventricular cells [18] and freshly isolated guinea-pig ventricular myocytes [148]. TRPM4 contributes to AP morphology, at least in mice, as the duration of left ventricular papillary APs was significantly smaller in TRPM4 KO mice compared with wild type ones [149]. Thereby, the increased driving force for Ca^2+^ entry via L-type Ca^2+^ current led to an increased contractility during β-adrenergic stimulation [149] involving the activation of adenylyl cyclase [150]. TRPM4 was involved in endurance training-induced beneficial cardiac remodeling [151]. In rats, the TRPM4 protein is clearly expressed in healthy adult ventricular myocytes [44] in contrast with the results of the Guinamard group. The reason for this difference can be the use of Wistar (or Wistar–Kyoto) rats in previous papers [43,147] while Sprague–Dawley rats have been used more recently [44,127]. Indeed, at least in mice, TRPM4 protein expression was about 80% higher in animals with a 129SvJ background versus a C57Bl/6N background [152]. Moreover, on the functional level, increased β-adrenergic inotropy was detected in global TRPM4-deficient 129SvJ mice, but the inotropic response was unaltered in C57Bl/6N mice with both global and cardiomyocyte-specific TRPM4 deletion [152].

Despite the conflicting data, TRPM4 inhibition can be a way of increasing inotropy. Recently a strategy using in vivo AAV9-RNAi-mediated silencing of cardiac TRPM4 was developed in the hope of increasing the cardiac contractility in animal models of cardiac failure, where a 90% reduction of TRPM4 protein expression was achieved in the adult mouse heart [153].

Further roles of TRPM4 in cardiac pathology will be discussed later in Section 3.7.

#### 2.2.9. The (Potential) Roles of TRPM4 in Other Tissues

TRPM4 mRNA was detected in both human and monkey colon tissues and in colonic smooth muscle cells [28]. TRPM4 might influence the resting membrane potential and determine basal excitability of colonic smooth muscle. Similarly, TRPM4 might be involved in setting the resting membrane potential and thereby regulating the basal tone of the ileal longitudinal smooth muscle of the mice [154].

Although TRPM4 is not principally regulated by temperature, calcium-activated monovalent cation currents were described in brown adipocytes [155,156]. TRPM4 can modulate calcium signaling in human adipose tissue-derived stem cells [157]. Human adipose tissue-derived stem cells express both mRNA and a protein of TRPM4, and its knockdown (KD) inhibits histamine-evoked lipid droplet accumulation and adipocyte differentiation [34]. Similarly, TRPM4 is required for rat dental follicle stem cell proliferation and survival [158]. Moreover, TRPM4 promotes adipocyte differentiation of rat dental follicle stem cells but inhibits osteogenesis [159]. A TRPM4 mRNA reduction was found during adipocyte differentiation in murine 3T3-L1 cells [160].

TRPM4 contributes to both mucin 2 and MUC5AC secretion in HT29-18N2 colonic cancer cells, where a Na^+^/Ca^2+^ exchanger 2 works in concert with TRPM4 [36]. Similarly, the amount of MUC5AC secretion was reduced after the blockade of NCX2 as well as upon the application of 9-phenanthrol in differentiated normal bronchial epithelial cells and tracheal cells from patients with cystic fibrosis [36]. TRPM4 can mainly be involved in stimuli-induced but not basal mucin secretion.

Among with many other TRP channels, TRPM4 was expressed in human osteoblastic phenotype-differentiated valve interstitial cells, where TRPM4 expression was higher in calcified tissues compared with control tissues [37].

TRPM4 mRNA was shown to be present in murine testis [87]; however, its presence was not detected previously [86]. In rats, TRPM4 mRNA was detected [161]. Western blot- and immunohistochemistry-detected TRPM4 in rat spermatogenic cells and spermatozoa suggests a yet unexplored role for TRPM4 in spermatogenesis [162].

Although TRPM4 mRNA was not found in murine lungs [86], two other studies reported TRPM4 mRNA in lung tissue [87] and freshly isolated and primary cultured type II cells from rat or healthy human lungs [163]. A calcium-activated monovalent cation current with typical properties including ion selectivity, Ca^2+^ dependence, and blockade by adenosine nucleotides could be mediated using TRPM4 and might reduce surfactant production.

In murine pancreatic acinar cells, a single channel current with properties of TRPM4 was present [164]. Later, TRPM4 mRNA was also detected; moreover TRPM4 was suggested to be involved in the negative feedback regulation of Ca^2+^ entry [165].

## 3. TRPM4 in Disease

### 3.1. TRPM4 in Skeletal Muscle

The role of TRPM4 is not widely known in skeletal muscle as only a few reports mention TRPM4 in relation to skeletal muscle, regardless of the fact that its presence was described in both murine [86,166] and human skeletal muscle tissue [8]. In the murine animal model of Duchenne muscular dystrophy, the TRPM4 mRNA level was much smaller in skeletal muscles of both 30 and 100 (but not 365)-day-old animals compared with those of healthy animals. This reduced TRPM4 expression could be only a secondary effect in response to the increased Ca^2+^ influx in dystrophy muscles [166]. Despite this, the contribution of TRPM4 to store operated Ca^2+^ entry and stretch-activated or background Ca^2+^ entry in muscle fibers is missing so far [167]. Moreover, the role of TRPM4 was only suggested as it might regulate membrane potential and, due to that, modulate the driving force for Ca^2+^ entry [168]. Alterations in TRPM4 splicing were reported in myotonic dystrophy 1 in both embryonic and adult muscle [169].

### 3.2. TRPM4 in Urinary Bladder

TRPM4 function in the smooth muscle of the urinary bladder made TRPM4 a potential target of overactive bladders [170]. TRPM4 expression was detected not only in the smooth muscle of the bladder itself [26,27,171] but also on the apical membrane of the umbrella cells of the urothelium [24,172]. In the detrusor muscle of both rats and guinea pigs, 9-phenanthrol dose-dependently inhibited spontaneous, carbachol-, KCl-, and nerve-evoked contractions [26,27,173]. The compound 9-phenanthrol was more effective, compared with glibenclamide, in reducing KCl-induced phasic contractions of the guinea pig detrusor muscle, but the two drugs were equally effective in reducing spontaneous contractions [25]. In case of the murine detrusor muscle, TRPM4 might play a role in setting the resting membrane potential, and, thereby, the basal tone can also influence the cholinergic signaling [154]. The detrusor muscle and mucosal cells increased their TRPM4 expression after spinal cord transection in mice, which led to spontaneous phasic activity [174]. As 9-phenanthrol greatly reduced this phasic activity, a potential role for TRPM4 in detrusor overactivity was suggested. Even more importantly, the human detrusor muscle, which also expressed TRPM4, was more sensitive to a 9-phenanthrol-induced reduction of contractility than the detrusor muscles of rats or guinea pigs [175]. Provence et al. suggested a spatial and functional coupling between TRPM4 channels and IP3 receptors in detrusor smooth muscle cells obtained from healthy humans [171]. Moreover, in guinea pigs, an overall reduction of the TRPM4 protein was reduced during aging and in line with that, the action of 9-phenanthrol (inhibition of the amplitude and muscle force of spontaneous and KCl-induced phasic contractions) was less pronounced in adult animals compared with juvenile ones [176].

### 3.3. The Role of TRPM4 in the Endothelium

TRPM4 can be involved in the function of endothelial cells. Ca^2+^-activated nonselective current was reported in Ea.hy926 cells, an endothelial cell line derived from human umbilical vein [177]. Similarly, a non-selective cation current was recorded in macrovascular endothelial cells derived from human umbilical veins, and the current was inhibited by various types of nitric oxide (NO) donors [178]. The ion channels responsible for the current might influence NO release by regulating the driving force for Ca^2+^ entry. The endothelial expression of TRPM4 was detected in a rat model of spinal cord injury [53]. In that model, preventing the in vivo expression of TRPM4 eliminated the secondary hemorrhage and greatly reduced lesion volume and, even more importantly, produced a substantial improvement in neurological function. Pharmacological (9-phenanthrol or glibenclamide) siRNA- or TRPM4-dominant negative mutant-mediated inhibition/suppression of TRPM4 protected the endothelium from lipopolysaccharide-induced endothelial cell death in human umbilical vein endothelial cells [179]. Similarly, the inhibition of TRPM4 expression in a rat ischemic stroke model improved the outcome, although this effect was only transient, in correlation with the ischemia-induced transient increase of TRPM4 expression [180]. Reduced TRPM4 expression induced by siRNA treatment led to decreased endothelial protein expression and, at the same time, increased expression of fibrotic and extracellular matrix markers [181]. The mechanism behind this effect was the increase of intracellular Ca^2+^ levels, the induction of TGF-β1 and TGF-β2 expression, and finally the nuclear translocation of the profibrotic transcription factor SMAD4. In conclusion, appropriate levels of TRPM4 may be beneficial and help to avoid endothelial dysfunction during inflammatory diseases [181]. TRPM4 increased but did not initiate H_2_O_2_-induced human umbilical vein endothelial cell depolarization and migration; therefore, it might also be involved in angiogenesis [182]. TRPM4 seems to be involved in endothelial cell functions and might become an important drug target.

### 3.4. The Role of TRPM4 in Cancer

TRPM4 was associated with several types of malignant diseases [39]. Usually overexpression of TRPM4 was reported in several types of malignancies, such as prostate cancer [183,184]; breast, cervical, and endometrial cancer [16,185,186,187]; diffuse large B cell lymphoma [188,189]; and acute myeloid leukemia [190]. TRPM4 protein overexpression was observed in colorectal cancer [191], but other studies reported either no differences in TRPM4 expression [192] or lower TRPM4 mRNA levels in colorectal cancer compared with normal tissue [193]. In the case of the urinary bladder, the TRPM4 protein was only detected in urothelium, and its expression was equal in cancer and control groups, which raises questions about its role in that type of malignancy [194]. TRPM4 might be useful as a diagnostic marker in the differentiation of eosinophilic renal tumors [195].

Endometrial carcinoma with reduced TRPM4 mRNA expression significantly correlated with poor prognosis, as well as overall and recurrence-free survival, making TRPM4 a prognostic factor [186,196]. Accordingly, TRPM4 silencing increased the cell viability and migration rate of the AN3CA endometrial cancer cell line [186]. On the contrary, in the cervical-cancer-derived cell line HeLa shRNA-mediated TRPM4 downregulation led to decreased cell proliferation, while overexpression of TRPM4 in a HEK293-derived cell line (T-REx 293) increased cell proliferation via the β-catenin pathway [197].

Two studies reported the involvement of TRPM4 in breast cancer, where its expression was increased on both the mRNA and protein levels [16,185]. Moreover, there was a correlation between increased TRPM4 protein expression and an estrogen response as well as Epithelial Mesenchymal Transmission (EMT) gene sets, resulting in worse clinic-demographical parameters [16]. Interestingly, the K^+^ channel tetramerization domain 5 protein was identified as a novel TRPM4-interacting protein, which enhances its Ca^2+^ sensitivity, promotes cell migration and contractility, and can also be used as a prognostic factor [185].

In colorectal cancer, as mentioned before, the expression pattern of TRPM4 is contradictory. The most recent study analyzed samples from 379 patients [191], while the earlier ones used either mRNA samples from 93 patients only [193] or 4 independent cultures of human colorectal (HT29) cell lines [192]. High TRPM4 protein expression in human colorectal tumor buds was associated with an epithelial–mesenchymal transition and infiltrative growth patterns. The highest level of TRPM4 protein expression was found in cells from late-stage metastatic cancer [191]. CRISPR/cas9 TRPM4 KO clones of colorectal carcinoma HCT116 cells showed a tendency toward decreased migration, invasion, cell viability, and proliferation, as well as exhibiting a shift in cell cycle. Stable overexpression of wild-type TRPM4, but not the non-conducting, dominant-negative TRPM4 mutant in CRISPR/cas9 TRPM4 KO clones, rescued the decrease in cell viability and cell cycle shift [191]. The importance of TRPM4 ion conductivity in modulation of viability and cell cycle shift makes TRPM4 a potential target in the treatment of colorectal cancer.

TRPM4 mRNA upregulation (together with the downregulation of other genes) in CD5+ subtypes of diffuse large B-cell lymphoma (DLBCL) patients was associated with a poorer prognosis compared with CD5− patients [189]. The TRPM4 protein was not detected in normal B cells within lymphoid tissues (reactive tonsil, lymph node, and appendix) but was observed in epithelial cells of reactive tonsils, epithelial luminal cells of hyperplastic prostates, endometrial glands, and distal tubules of kidneys [188]. Out of 189 DLBCL cases, 26% exhibited 10–100% TRPM4-positive tumor cells, and the high TRPM4 expression in activated B cell-like DLBCL subtype was associated with a reduced overall and progression-free survival [188]. TRPM4 expression also increased in those acute myeloid leukemia patients and acute myeloid leukemia cell lines where the MLL gene (a methyltransferase for histone H3 lysine 4) was rearranged [190]. TRPM4 KD by siRNA in MLL-rearranged cell lines inhibited proliferation and cell cycle progression through the AKT/GLI1/Cyclin D1 pathways. The transcription factor HOXA9 was found to be responsible for the upregulation of TRPM4 expression by binding to its promoter in MLL rearranged cell lines [190]. The up-regulation of TRPM4, the only surface protein among up-regulated gene products in acute myeloid leukemia cell lines made TRPM4 a potential novel therapeutic target [198].

TRPM4 is well studied in prostate cancer. TRPM4 mRNA was present in normal human [8] and rat prostate tissue [199]. Higher levels of TRPM4 mRNA were found in prostate cancer upon comparing a pool of 11 normal and 13 precancerous or cancerous prostate tissue-derived data using digital libraries [200]. TRPM4 can be a driver gene of androgen-independent prostate cancer in vitro [201]. The TRPM4 protein was strongly expressed in histological samples of 20 prostate cancer patients, but weak or no expression was seen in benign prostatic hyperplasia tissues [202]. Similarly, in a study of 614 patients, significantly higher TRPM4 staining intensity was found in glands of prostate cancer compared with benign glands [203]. When TRPM4 expression was high, i.e., equal to or above the median histological score, the risk of biochemical recurrence after radical prostatectomy was increased [203]. Another study of 210 prostate cancer patient tissues also demonstrated a positive association between TRPM4 protein expression and local/metastatic progression [183]. On the contrary, increased TRPM4 mRNA expression was only detected in those prostate cancer samples with a Gleason score higher than 7, which is more likely to spread [17]. Both healthy prostate (hPEC) and androgen-insensitive prostate cancer cell lines DU145 and PC3 possessed large TRPM4-mediated Na^+^ currents [202]. Significantly, store-operated calcium entry increased after siRNA targeting of TRPM4 in hPEC and DU145 cells. TRPM4 KD led to reduced migration but not proliferation of DU145 and PC3 cells, suggesting a role for TRPM4 in cancer cell migration and marking TRPM4 as an anticancer therapeutic target [202]. The mechanism of action by which TRPM4 alters the progression of prostate cancer was also described [204]. Decreasing TRPM4 levels in PC3 cells reduced both total and nuclear localized β-catenin protein levels due to an increase in β-catenin degradation. TRPM4 overexpression in lymph node carcinoma of the prostate cell line increased the total levels of β-catenin, proposing a role for TRPM4 channels in β-catenin oncogene signaling and again enforcing TRPM4 as a new potential target for future therapies in prostate cancer [204]. Not only the proliferation of prostate cancer cells, but also their migration/invasion capability, were influenced by TRPM4. Reducing TRPM4 expression in PC3 cells decreased their migration/invasion capability, possibly due to the reduction in the expression of Snail1, a canonical epithelial to mesenchymal transition (EMT) transcription factor. Inversely, overexpression of TRPM4 in lymph node carcinomas of the prostate cells resulted in increased levels of Snail1 and an increase in its migration potential [17]. Upregulation of miR-150 reduced TRPM4 expression in PC3 cells and resulted in inactivation of the β-catenin signaling pathway, leading to beneficial actions on cancer cells both in vitro (suppression of EMT, proliferation, migration, and invasion) and in vivo (restrained tumor growth and metastasis) [205]. Similarly to previous results, stable CRISPR/Cas9-mediated TRPM4 KO led to lower proliferation, migration, and viability, as well as to reduced cell adhesion and a rounder shape of DU145 cells [183]. Interestingly, despite causing partial inhibition of TRPM4 currents in DU145 cells, the novel inhibitors CBA, NBA, and LBA did not evoke any TRPM4-specific effect in the cellular assays, which questions the role of TRPM4 ion conductivity in cancer hallmark functions in prostate cancer [183].

As mentioned above, the role of TRPM4 in cancer is often based on observations where it exhibits a different expression compared with control tissues; sometimes the correlation between the amount of TRPM4 expression and the prognosis and/or the post-treatment reoccurrence of the disease was studied [190,191,196,203]. Several studies described the molecular mechanisms by which TRPM4 influences these diseases. One mechanism involves TRPM4 activation, which changes the membrane potential, thereby influencing Ca^2+^ entry by changing the driving force of the Ca^2+^ influx [202,204]. TRPM4 can co-localize with focal adhesion proteins in mouse embryonic fibroblasts [206] and in commonly used cell lines (HEK and COS-7 cells) [207] to regulate focal adhesion turnover, a process important for cell migration and invasion. TRPM4 channel activity is greatly enhanced by ATP depletion and increases in the intracellular Ca^2+^ level; both changes are associated with hypoxia, a condition commonly present in cancer cells. Moreover, TRPM4 can play a key role in stemness mediation [208]. Cancer stem cells play roles in chemoresistance, tumor recurrence, and metastasis in breast cancer. TRPM4 inhibition reduced stemness properties of breast cancer stem cells in vitro, therefore TRPM4 can be a novel therapeutic target [208].

### 3.5. The Importance of TRPM4 in Central Nervous System (CNS) Pathophysiology

The importance of TRPM4 is greatest in the case of cerebral physiology highlighted by the following: the high number of reviews, the co-expression of TRPM4 with Sulfonylurea Receptor 1 (SUR1) only in the case of pathological conditions, and the very promising actions of TRPM4 inhibition by either pharmacological (using glibenclamide) or other strategies in both experimental models and human CNS pathologies. Some human studies using intravenous glibenclamide administration have already been completed, some are currently enrolling patients. Recent reviews presented not only clinical trials using glibenclamide [209,210], but also the expression and role of SUR1-TRPM4 in CNS injury animal models and in human conditions associated with cerebral edema [210,211]. Apart from TRPM4, other possible drug targets of cerebral edema were also detailed [211,212]. Among these targets is the SUR1-TRPM4 channel complex (and also its complex with the aquaporin4 water channel protein) in astrocytes, which can contribute to swelling [213] but not cell death [214]. Other mechanisms leading to astrocyte swelling were also discussed [215]. It seems that astrocytes are less sensitive to hypoxic-injury-induced oncotic cell death compared with neurons and vascular endothelial cells [216].

SUR1, a per se inactive regulatory subunit of ion channels, can associate with TRPM4 and also with type 6.2 of inward rectifier K^+^ channel (Kir) to form SUR1-TRPM4 (previously called Sur1-NSCCa-ATP) and adenosine triphosphate-dependent K^+^ (K_ATP_) channels, respectively [217]. The functional consequence is exactly the opposite in the case of the two channels. Upon activation, SUR1-TRPM4 leads to depolarization, whereas during the activation of K_ATP_ channels, it results in the hyperpolarization of cells [218]. In physiological conditions, only K_ATP_ channels are present in certain neurons and microglia [219]. On the contrary, SUR1-TRPM4 does not exist normally in the central nervous system, SUR1 was shown to be upregulated in pathological conditions in all cell types (neurons, astrocytes, oligodendrocytes, and endothelium) of the neurovascular unit in the penumbra but not in the necrotic region [220,221,222]. Both the mRNA (Abcc8) and the protein of SUR1 upregulated without the upregulation of Kir6.2 protein or its mRNA (Kcnj11) [220,223]. Recently, the SUR1 content of the cerebrospinal fluid increased variably in some (but not all) pediatric patients with traumatic brain injuries (TBI) [224]. Based on these observations, the other partner of SUR1, TRPM4, was also likely to be expressed. Indeed, this was confirmed on both mRNA and protein levels, not only in animal models [225,226], but also in brain specimens from 15 patients who died within a month of the onset of focal cerebral ischemia [226,227]. TRPM4 expression was also higher after the noxious stimulation of rats in the previously contusion-injured spinal cord [228].

SUR1-TRPM4 channel expression has especially great importance due to the lack of other useful targets in the treatment of cerebral edema which accompanies several CNS pathologies (stroke, TBI, and subarachnoid hemorrhage). In the case of stroke management, many potential targets such as N-methyl-d-aspartate and a-amino-3-hydroxy-5-methyl-4-isoxazole propionate receptor antagonists, clomethiazole, antioxidants, citicoline, nitric oxide, immune regulators, therapeutic hypothermia, magnesium, albumin, glibenclamide, and uric acid were discussed [229]. Earlier, potential therapeutic targets for CNS injury management included the inhibition of acid sensing ion channels [230], the Na-K-2Cl symporter with the functionally coupled Na-K ATPase [231], Na-Ca exchanger [232], and vasopressin receptor [233]. A recent review summarizes all ion channels and transporters which can have a proposed role in mediating cerebral edema in acute ischemic stroke and TBI [211]. Moreover, in intracerebral hemorrhage, the potential targets for reducing secondary injury mechanisms were summarized [234]. These previous reviews also highlight the need for a good target and numerous attempts to find a suitable target.

After the CNS injury, the secondary consequences usually culminate in cerebral edema. Only one approved pharmacological treatment exists against the edema: the application of a recombinant tissue plasminogen activator (tPA). Apart from recombinant tPA, decompressive craniectomy can be applied. A craniectomy can reduce the intracranial pressure, which otherwise reduces cerebral blood flow and results in herniation, i.e., life-threatening consequences of high intracranial pressure [235,236]. Surgical intervention, however, is only a symptomatic treatment, and it would be desirable to avoid progression and development of the rise in intracranial pressure. Despite the benefit of a craniectomy, glibenclamide treatment was even more effective in a rat model malignant stroke [221]. Using recombinant tPA can be effective, but it is limited by the short therapeutic window and the possible risk of hemorrhagic conversion [237]. Therefore, tPA is only applied in approximately 20% of patients [238]. Among the many ion channel and transporter targets, SUR1-TRPM4 had the longest therapeutic window [232]. Glibenclamide application was effective upon administration even 10 h after the event in a clinically relevant rat stroke model [239]. Glibenclamide effectively reduced CNS injury-induced harmful consequences in rat [240,241,242,243,244] and mouse animal models [245,246] of TBI from independent labs. Glibenclamide application was also beneficial in stroke models in adult rats [217] and to a certain extent (providing some long-term neuroprotective effect in moderate but not severe hypoxia–ischemia) in neonatal rats too [247]. Glibenclamide administration exerted a beneficial effect on subarachnoid [248,249], but not in intracerebral, hemorrhages [250,251]. In the case of an intracerebral hemorrhage model with aged rats, glibenclamide treatment improved neurological outcomes and ameliorated neuroinflammation [252]. Glibenclamide treatment exerted beneficial actions in a model of hemorrhagic encephalopathy of prematurity [253], just as it did in inflammation-associated conditions of the CNS [248,249,254] and other organs (respiratory, digestive, urological, and cardiac) [255]. Moreover, glibenclamide application was beneficial in HIV infection in vitro [256]. In a mouse model of peripheral nerve injury, glibenclamide-induced inhibition of the newly expressed SUR1 in astrocytes reduced neuropathic pain [257]. Furthermore, glibenclamide treatment reduced the edema and thereby improved the glymphatic flow in status post epilepticus [258]. Likewise, glibenclamide improved the outcome in murine experimental autoimmune encephalomyelitis [54]. It must be noted that the beneficial effect of glibenclamide in stroke models might also be mediated by the blockade of K_ATP_ channels on rat CA1 pyramidal neurons in vitro [259] or by the in vivo blockade of microglial K_ATP_ channels in rats [222,260,261]. This option was questioned later, as specific antisense oligonucleotides targeted against SUR1 or TRPM4, but not Kir6.1 or Kir6.2 (partners of SUR1 in forming K_ATP_ channels), significantly reduced hemispheric swelling in rats in post-ischemic tissues showing co-assembly of SUR1-TRPM4 heteromers [225].

The benefit of glibenclamide was summarized by several publications in stroke [262,263] and spinal cord injury [264] in humans. In a retrospective study, the outcome of stroke in those type 2 diabetic patients continuously on glibenclamide treatment was better compared with those on other medications or suspended glibenclamide treatment [265]. Symptomatic hemorrhagic transformation was found in 0 and 11% of these acute ischemic stroke patients with and without sulfonylurea treatment, respectively [266]. On the contrary, sulfonylurea use before stroke did not influence the outcome compared with other antidiabetic medications [267]. Similarly, while diabetic patients on sulfonylurea treatment showed lower peripheral edema volumes after basal ganglia hemorrhage, their clinical outcome did not improve [268]. Moreover, it was reported that type 2 diabetic patients with sulfonylureas had higher odds ratios for stroke morbidity than those who received comparator drugs [269]. Although glibenclamide applied orally in diabetic patients, application of intravenous glibenclamide could provide more stable plasma levels; it was applied in stroke patients, where it appeared to reduce several surrogate markers of vasogenic edema [270]. Intravenous glibenclamide was well tolerated and free of adverse effects, as reported in two studies GAMES (Glyburide Advantage in Malignant Edema and Stroke)-Pilot and GAMES-RP [271,272] and improved survival in participants ≤70 years of age with large hemispheric infarction [273]. Currently the CHARM (Cirara in large Hemispheric infarction Analyzing modified Rankin and Mortality) trial is a randomized, double-blind, placebo-controlled, parallel-group, multicenter, Phase 3 study which is ongoing to evaluate the efficacy and safety of intravenous glibenclamide for severe cerebral edemas following large hemispheric infarction. Intravenously administered glibenclamide was safe and well tolerated in case of a TBI pilot in a small Phase 2, three-institution, randomized placebo-controlled trial [274]. The treatment reduced hemorrhage volumes and the increase of lesion volumes (hemorrhage plus edema) was much smaller when compared with placebo [274]. ASTRAL (Antagonizing SUR1-TRPM4 to Reduce the progression of intracerebral hematoma and edema surrounding Lesions) is another currently running multicenter, double-blind, multidose, placebo-controlled, randomized, parallel-group, Phase 2 study enrolling only contusion-TBI patients. Jha et al. recently reviewed in detail the clinical and preclinical studies using not only intravenous but also oral glibenclamide in TBI [210].

The effectivity of glibenclamide treatment seems convincing given the facts that: (1) the drug much more potently inhibits SUR1-TRPM4 channels than TRPM4 channels [275]; (2) SUR1-TRPM4 channels are upregulated only in injured CNS tissues [220,249]; (3) glibenclamide treatment has a long therapeutic window [239]; and (4) glibenclamide treatment is free of major side effects (there is a small risk of hypoglycemia, which can be well managed or greatly reduced by intravenous application) [209]. Due to the weak acidic nature of glibenclamide, it is mainly accumulated in the acidic areas of the body [276], which further reduces its potential side effects, as TRPM4 can be present in neurons of various brain areas (see above). Moreover, the inhibitory potential of glibenclamide increases with pH reduction [220]. Fortunately, glibenclamide preferentially inhibits SUR1, as other potential targets such as SUR2 are one to two orders of magnitude less sensitive [277].

In addition to the abovementioned glibenclamide, but the equally effective glimepiride was beneficial—at least in mice with acute ischemic stroke [278]. A previously often-used TRPM4 inhibitor, flufenamic acid, also improved functional recovery in a murine spinal cord injury model [279]. Selective gene suppression of ABCC8 (a gene of SUR1) or TRPM4 applied in spinal cord injury models showed a beneficial effect similar to glibenclamide application [53,223]. Along with that, an obtained KO of either ABCC8 or TRPM4 was protective and prevented capillary fragmentation, halted progressive hemorrhagic necrosis, and prevented the spread of the hemorrhagic contusion [53,223]. TRPM4 KO also reduced cerebral edema and neuronal injury in a murine model of status epilepticus [52]. Antisense oligodeoxynucleotides directed against ABCC8 prevented capillary fragmentation and further accumulation of blood in TBI [241]. These studies emphasize the importance of SUR1-TRPM4 in the pathomechanism of edema formation after CNS injuries.

The role of SUR1-TRPM4 was detailed in several reviews [210,230,280,281]. Briefly, the Na^+^ influx through newly expressed SUR1-TRPM4 (and other) channels in neurons and astrocytes leads to water influx driven by osmotic gradient in cytotoxic edema. In astrocytes aquaporin-4 water channels can also be involved in SUR1-TRPM4 protein complexes and contribute to cell swelling [213]. Ionic edema formation is facilitated by ion channels, transporters, and newly expressed SUR1-TRPM4 channels on endothelial cells. In the case of vasogenic edema, water and plasma proteins also leave capillaries due to the disruption of the blood–brain barrier. Tight-junction degradation and endothelial cell damage are the reasons for blood–brain barrier damage. Upon complete breakdown of the blood–brain barrier, hemorrhagic conversion occurs, leading to further worsening of the situation. Jha et al. reviewed the molecular mechanisms leading to edema formation [210]. Upstream mechanisms involved in SUR1-TRPM4 upregulation are the hypoxia-inducible factor-1 α, specificity protein 1 [282], and tumor necrosis factor α [241]. Lipopolysaccharide-induced neuroinflammation activated the toll-like receptor 4 and led to de novo upregulation of SUR1-TRPM4. The SUR1-TRPM4-induced depolarization activated calcineurin via induction of low-amplitude repetitive Ca^2+^ oscillations. Calcineurin dephosphorylates NFATc1, eventually leading to upregulation of nitric oxide synthase-2 mRNA and protein, as well as increased nitric oxide production [283]. Nitric oxide can be converted to harmful peroxynitrite, which can induce protein radical formation [284]. Glibenclamide can evoke a direct antioxidant effect [285]. Blood–brain barrier damage can also occur due to oncotic endothelial cell death [281], mechanisms involving Zonula Occludens-1 disfunction/disruption [245,248], and tPA-induced matrix metalloproteinase-9 production [286].

It appears that SUR1-TRPM4 is indeed a very promising target in the pathological conditions of the CNS. Moreover, serum levels of both SUR1 and TRPM4 can be potential diagnostic markers in the future, at least in aneurysmal subarachnoid hemorrhage [287]. Moreover, a recent study found an association between the progression of intraparenchymal hemorrhages after TBI and 4-4 single-nucleotide variants of ABCC8 and TRPM4 genes [288].

TRPM4 can contribute to glutamate excitotoxicity as TRPM4 and NMDA receptors in extrasynaptic locations are in physical interaction [289]. The NMDA receptor/TRPM4 complex formation is required for excitotoxicity and is mediated by a 57-amino acid intracellular domain and a highly conserved stretch of 18 amino acids of TRPM4 and NMDA receptor (GluN2A and GluN2B, respectively). In mouse models of stroke and retinal degeneration, the inhibition of the TRPM4-interacting domain, called TwinF, by small molecular inhibitors proved to reduce excitotoxicity without impairing the function of synaptic NMDA receptors [289].

For a comprehensive review on the role of sulfonylurea receptor 1 in central nervous system injury see the work of Jha et al. [290].

### 3.6. The Pathophysiological Role of TRPM4 in the Skin

Two TRPM4 missense mutations leading to increased activation gating and enhanced Ca^2+^ sensitivity were reported to cause an autosomal dominant form of progressive symmetric erythrokeratodermia [35]. In keratinocytes overexpressing those TRPM4 mutants, enhanced proliferation and up-regulation of proliferation and differentiation markers were detected.

### 3.7. Involvement of TRPM4 in Cardiac Disorders

#### 3.7.1. The Role of TRPM4 in Cardiac Hypertrophy and Heart Failure

Although the causative link between TRPM4 mutations and conduction problems is clearly established, TRPM4 can also be involved in other cardiac problems [291,292] such as cardiac hypertrophy [293,294]. For instance, higher TRPM4 expression was shown in hypertrophied ventricular cells obtained from the hearts of spontaneously hypertensive rats compared with control Wistar–Kyoto rats [43]. Moreover, in the case of dedifferentiated cultured rat ventricular cardiomyocytes with similar characteristics to hypertrophied cells, TRPM4 could be detected, while it was absent in freshly isolated ventricular cells [146]. TRPM4 (together with other mechanisms) can contribute to arrhythmias (by delayed afterdepolarization (DAD) generation), especially in Ca^2+^ overloaded cells [295].

On the contrary, TRPM4 has a protective role in hypertensive hypertrophy and heart failure in mice as (1) TRPM4 KO mice are hypertensive due to an increased catecholamine release [77]; (2) their β-adrenergic inotropic response in ventricular myocardium was increased compared with wild type counterparts [149]; (3) angiotensin II-induced cardiac hypertrophy was more pronounced in KO vs. wild type mice [296]; and (4) left ventricular eccentric hypertrophy with an increase in both wall thickness and chamber size was detected in the adult TRPM4 KO mice, compared with wild type littermate controls [117]. TRPM4 expression in right ventricles was reduced in a rat model of pulmonary hypertension, and right ventricular hypertrophy was more severe when TRPM4 was absent (TRPM4 KO) compared with wild type animals [111].

In contrast, TRPM4 can act as a positive regulator of pressure-overload induced left ventricular hypertrophy as selective deletion of TRPM4 in mouse cardiomyocytes resulted in an approximately 50% reduction of left ventricular hypertrophy induced by transverse aortic constriction [297].

In the case of humans, TRPM4 mRNA levels were 65% higher in left ventricular tissue samples of patients with end-stage heart failure (NYHA III–IV), compared with those obtained from healthy control subjects [298]. There was, however, no correlation between the TRPM4 mRNA level and either gender or the clinical, biochemical, and functional parameters of the heart. Nevertheless, TRPM4 protein expression was also higher (about twice as much) in human left ventricular tissue samples of heart failure patients compared with samples from healthy donor hearts [299]. For this larger tissue expression, the higher TRPM4 currents in cardiac fibroblasts could be responsible, although the contribution of ventricular myocytes cannot be excluded, as those cells were not studied [299].

#### 3.7.2. The Role of TRPM4 in Ischemia-Reperfusion Injury

Unlike with cardiac hypertrophy induced by hypertension, TRPM4 mRNA remained unchanged during both 25 min of ischemia and the following 40-min-long reperfusion in rats [300]. Nevertheless, the inhibition of TRPM4 reduced hypoxia-reperfusion-induced EAD formation in murine isolated right ventricular muscle, especially during reperfusion [301]. The frequency of EADs was greatly and dose-dependently abolished by the TRPM4 inhibitor 9-phenanthrol (and flufenamic acid) [301]. Similarly, pretreatment with 9-phenanthrol dramatically improved contractile function recovery during reperfusion of rat hearts and significantly reduced the infarcted area size [302]. Furthermore, in H9c2 cardiomyocytes, H_2_O_2_-evoked reactive oxygen species-induced injury was reduced not only by 9-phenanthrol pretreatment but also TRPM4 silencing [44]. The mechanism behind the harmful action of TRPM4 in that cell line might involve the reduction of mitochondrial membrane potential and intracellular ATP levels (as well as the intracellular Ca^2+^ overload), as those were absent in TRPM4 KO H9c2 cells [303]. TRPM4 can be responsible for the generation of not only EADs, but also DADs, a possible mechanism explaining the higher incidence of death upon isoprenaline-induced β-adrenergic stimulation in wild type animals compared with those of TRPM4 KO in a murine ischemic heart failure model [304]. Of note, basal cardiac parameters were not different between the wild type and KO mice. Computational modeling also highlighted the role of TRPM4 in EAD generation [125]. In vivo TRPM4 overexpression increased the vulnerability to premature ventricular ectopic beats during exercise-induced β-adrenergic stress [305]. In contrast, TRPM4 was beneficial; moreover, it was essential for survival in a mouse model of myocardial infarction [306]. Unlike unaltered TRPM4 mRNA expression in rats [300], murine TRPM4 expression increased at both the mRNA and protein levels in a mouse model of myocardial infarction [306]. Murine TRPM4 KO animal models are summarized in Table 4.

## 4. Conclusions

Although TRPM4 was first described in the beginning of the 21st century, reports about a Ca^2+^-activated non-specific cationic current in various tissues were published long before that. Since then, a tremendous amount of knowledge has been accumulated about TRPM4, but there are still questions to be answered. For instance, results are not always congruent when we compare experimental data obtained from KO animals with data using TRPM4 inhibitors. Nevertheless, promising results show the potential of TRPM4 inhibition for the treatment of patients with various conditions of the central nervous system. TRPM4 might also be a therapeutic target for other conditions in the future.

## Figures and Tables

**Table 1:** Summary of murine non-cardiac TRPM4 KO animal models.

Properties of Animal Model	Targeting Strategy	Impact on Protein	KO Animals Showed:	Possible Link to Human Diseases	Conclusion	References
2–6 month-old C57BL/6 TRPM4 KO mice	Cre-loxP–mediated recombination	No TRPM4 protein	less response to different taste stimuli	?	TRPM4 (and TRPM5) is important in the transduction of bitter, sweet, and umami tastes	[51]
6–8 week-old, male C57BL/6J TRPM4 KO mice with post status epilepticus	not stated	not stated	less water content, cerebral edema, reduced mortality, and cognitive deficit	?	TRPM4 may represent a new target for improving outcomes after status epilepticus	[52]
24–28 g weight C57BL/6 TRPM4 KO mice with unilateral spinal cord injury	Cre-loxP–mediated recombination	No TRPM4 protein	reduction in spinal cord injury lesion volume, substantial improvement in neurological function	spinal cord injury	TRPM4 upregulates and initiates secondary hemorrhage in spinal cord injury	[53]
TRPM4 KO mice	Cre-loxP–mediated recombination	No TRPM4 protein	increased stimulated Ca^2+^ entry and histamine, leukotrienes and tumor necrosis factor release in mast cells	anaphylactic response	TRPM4 channels are critical regulators of Ca^2+^ entry into mast cells	[15]
6–10 week-old C57BL/6J TRPM4 KO mice with experimental autoimmune encephalomyelitis	Cre-loxP–mediated recombination	No TRPM4 protein	Reduced axonal and neuronal injury and disease severity	multiple sclerosis	TRPM4 contributes to inflammatory effector mechanisms	[54]
8–12 week-old 129/B6 TRPM4 KO mice	not stated	not stated	increased mortality and reduced phagocytic activity of monocytes and macrophages	sepsis	TRPM4 is protective in cecal ligation and puncture-induced sepsis model	[55]
TRPM4 KO mice	Cre-loxP–mediated recombination	No TRPM4 protein	reduced dinitrophenylated human serum or stem cell factor-induced migration	inflammation reactions	TRPM4 is involved in the migration of bone-marrow-derived mast cells	[56]

**Table 2:** Summary of rat TRPM4 KO animal models.

Properties of Animal Model	Targeting Strategy	Impact on Protein	KO Animals Showed:	Possible Link to Human Diseases	Conclusion	References
7–10 week-old, male TRPM4 KO rats	514 bp deletion, complete removal of exon 18, intron 18–19, and a piece of exon 19	No channel pore (removal of TM3, TM4, and a piece of TM5 regions)	deficit in spatial working and reference memory	?	TRPM4 contributes to hippocampal synaptic plasticity and learning	[78]
7–10 week-old, male TRPM4 KO rats	same as above	same as above	significantly delayed and less pronounced decline in baseline BOLD signals	?	TRPM4 channels can be involved in mediating baseline BOLD shifts	[79]
14–15 week-old male Sprague-Dawley TRPM4 KO rats with RV pressure load	same as above	same as above	no change and increased RV hypertrophy without and with RV pressure load, respectively	RV remodeling in patients with pulmonary hypertension	complete deletion of TRPM4 expression aggravates RV hypertrophy	[111]

RV: right ventricular.

**Table 3:** TRPM4 mutations involved in cardiac conduction disorders.

Amino Acid Replacement	Condition	Effect	Mechanism	References
E7K	PFHB1	G.o.F	reduced endocytosis (SUMOylation problem) increased PIP2, voltage and Ca^2+^ sensitivity	[129,131,132]
R164W	ICCD	G.o.F	reduced endocytosis (SUMOylation problem)	[11]
A432T	ICCD	G.o.F	reduced endocytosis (SUMOylation problem)	[11,12]
A432T	BrS	?	?	[139]
A432T and A432T + G582S	congenital and childhood AVB	L.o.F	smaller current	[133]
G582S	congenital and childhood AVB	G.o.F	no change in SUMOylation	[133]
G582S	BrS	?	?	[139]
G582S	incomplete RBBB	?	?	[12]
G844D	ICCD	G.o.F	increased Ca^2+^ sensitivity	[11,12]
G844D	BrS		mutation in SCN5A too	[140]
G844D	LQTS	?	?	[143]
I376T	PFHB1	G.o.F	larger current	[134]
Q854R	CHB	G.o.F	?	[135]
A101T and A101T+P1204L	CHB	L.o.F	?	[135]
A101T	BrS	?	?	[12]
S1044C	CHB	L.o.F	?	[135]
R819C	AVB	?	?	[136]
G858A	left ventricular noncompaction and progressive cardiac conduction defects	L.o.F	?	[137]
T873I	BrS	G.o.F	?	[139]
L1075P	BrS	G.o.F	?	[139]
P779R	BrS	L.o.F	?	[139]
K914X	BrS	L.o.F	?	[139]
Y103C	BrS	?	?	[12]
R252H	BrS	?	?	[12]
V441M	LQTS	L.o.F	?	[143]
R499P	LQTS	L.o.F	?	[143]
T160M polymorphism	LQTS		with KCNQ1 G219E mutation	[144]
P970S	incomplete RBBB	?	?	[12]
Q131H	incomplete RBBB	?	?	[12]
Q213R	AVB	?	?	[12]
K914R	AVB	?	?	[12]
combination of two heterozygous TRPM4 null mutations	complete RBBB	L.o.F	?	[141]

AVB: atrioventricular block; BrS: Brugada syndrome; CHB: complete heart block; G.o.F: gain of function; ICCD: isolated cardiac conduction disease; L.o.F: loss of function; LQTS: long QT syndrome; PFHB1: progressive familial heart block type I; RBBB: right bundle branch block.

**Table 4:** Summary of murine cardiac TRPM4 KO animal models.

Properties of Animal Model	Targeting Strategy	Impact on Protein	KO Animals Showed:	Possible Link to Human Diseases	Conclusion	References
12 and 32 week-old, male TRPM4 KO mice	not stated	not stated	LV eccentric hypertrophy with an increase in wall thickness and chamber size, smaller ventricular cells and probable hyperplasia, shorter atrial APs	cardiac conduction disorders	TRPM4 regulates conduction and cellular electrical activity	[117]
6–14 week-old TRPM4 KO mice	not stated	not stated	no change in rate of isolated right atria but much smaller effect of 9-phenanthrol, shorter right atrial APs	Sinus node dysfunction	TRPM4 contributes to pacemaking	[40]
4–6 week-old, female C57/BL6JRj TRPM4 KO mice	not stated	not stated	20–30% shorter atrial APs and much smaller effect of 9-phenanthrol	?	TRPM4 contributes to atrial action potential	[42]
2-month-old, male C57/BL6JRj TRPM4 KO mice	not stated	not stated	higher atrial diameter and triggered arrhythmia hyperaldosteronemia-salt prolonged left atrial AP	?	TRPM4 is involved in aldosterone-induced atrial AP shortening and arrhythmias	[119]
18–19 week-old, male 129SvJ TRPM4 KO mice	Cre-loxP–mediated recombination	No TRPM4 protein	increased cardiac contractility under β-adrenergic stimulation	?	TRPM4 can be involved in β-adrenergic stimulation induced ventricular inotropic effect	[152]
18–19 week-old, male C57Bl/6N global and cardiomyocyte-specific TRPM4 KO mice	cardiac-specific deletion of exon 15 and 16	cardiac-specific absence of the first transmembrane domain	unaltered inotropic response	?	TRPM4 expression is higher in 129SvJ versus C57Bl/6N mice	[152]
21 week-old C57Bl6/N TRPM4 KO mice with severe ischaemic HF	Cre-loxP–mediated recombination	No TRPM4 protein	unaltered or increased contractility in basal conditions and during beta-adrenergic stimulation, respectively	ischaemic HF	TRPM4 can worsen survival and reduce beta-adrenergic cardiac reserve in ischaemic HF	[304]
129/SvJ TRPM4 KO mice	Cre-loxP–mediated recombination	No TRPM4 protein	increased β-adrenergic inotropic response, shorter ventricular AP	?	TRPM4 can be a novel determinant of β-adrenergic stimulation induced ventricular inotropic effect	[149]
3–8 month-old, male 129/SvJ TRPM4 KO mice	Cre-loxP–mediated recombination	No TRPM4 protein	increased systolic and diastolic blood pressure, epinephrine concentration and urinary excretion of catecholamine metabolites	hypertension	TRPM4 limits catecholamine release and can prevent sympathetic tone-induced hypertension	[77]
3–6 month-old, male C57BL/6 N Cardiac-specific TRPM4 KO mice	cardiac-specific deletion of exon 15 and 16	cardiac-specific absence of the first transmembrane domain	increased hypertrophic growth and store-operated calcium entry after chronic angiotensin treatment	cardiac hypertrophy	TRPM4 contributes to the development of pathological hypertrophy	[296]

AP: action potential; HF: heart failure; LV: left ventricular.

## Data Availability

Data sharing not applicable.

## Abbreviations

AP	action potential
ATP	adenosine triphosphate
DAD	delayed afterdepolarization
DLBCL	diffuse large B-cell lymphoma
EAD	early afterdepolarization
HEK	human embryonic kidney
hPEC	human prostate epithelial cells
IP3	inositol 1,4,5-trisphosphate
I(NSC_Ca_)	Ca^2+^-activated non-specific cationic current
K_ATP_	adenosine triphosphate-dependent K^+^
KD	knock-down
KO	knock-out
NFATc1	nuclear factor of activated T-cells
NMDA	N-methyl-d-aspartic acid
NO	nitric oxide
NSC_Ca_	Ca^2+^-activated non-specific cationic channel
PIP2	phosphatidylinositol 4,5-bisphosphate
PKC	Protein Kinase C
tPA	tissue plasminogen activator
siRNA	small-interfering RNA
SUR1	Sulfonylurea Receptor 1
TBI	traumatic brain injury
TGF-β	transforming growth factor β
TRP	transient receptor potential
TRPA	transient receptor potential ankyrin
TRPC	transient receptor potential canonical
TRPM	transient receptor potential melastatin
TRPML	transient receptor potential mucolipin
TRPP	transient receptor potential polycystin
TRPV	transient receptor potential vanilloid
